# Stability and Antioxidant Activity of Hydro-Glyceric Extracts Obtained from Different Grape Seed Varieties Incorporated in Cosmetic Creams

**DOI:** 10.3390/antiox11071348

**Published:** 2022-07-10

**Authors:** Yara Salem, Hiba N. Rajha, Diana Franjieh, Israa Hoss, Maria Letizia Manca, Maria Manconi, Ines Castangia, Matteo Perra, Richard G. Maroun, Nicolas Louka

**Affiliations:** 1 Centre d’Analyses et de Recherche, Unité de Recherche Technologies et Valorisations Agro-Alimentaire, Faculté des Sciences, Université Saint-Joseph de Beyrouth, P.O. Box 17-5208, Beirut 1104 2020, Lebanon; yara.salem1@net.usj.edu.lb (Y.S.); diana.franjieh@gmail.com (D.F.); israa.hoss@net.usj.edu.lb (I.H.); richard.maroun@usj.edu.lb (R.G.M.); nicolas.louka@usj.edu.lb (N.L.); 2 Ecole Supérieure d’Ingénieurs de Beyrouth (ESIB), Université Saint-Joseph de Beyrouth, CST Mkalles Mar Roukos, Beirut 1107 2050, Lebanon; 3 Department of Life and Environmental Sciences, University of Cagliari, Via Ospedale n.72, 09124 Cagliari, Italy; manconi@unica.it (M.M.); ines.castangia@unica.it (I.C.); matteo.perra@unica.it (M.P.)

**Keywords:** grape seeds, hydro-glyceric extracts, natural antioxidant, skincare products, cosmetics

## Abstract

Grape seeds are agro-industrial by-products, which if improperly managed, may be responsible for socioeconomic and environmental problems. Nevertheless, it is possible to effectively valorize them by means of extraction of the bioactive compounds, especially the antioxidant phenolic molecules, using a safe, green, and environmentally-friendly extractive medium (i.e., hydro-glyceric solution). In the present study, the extraction was performed using seeds from two Lebanese varieties, Obeidi and Asswad Karech, and three international varieties, Marselan, Syrah, and Cabernet Franc. The type and amount of phenolic compounds were identified by High-Performance Liquid Chromatography (HPLC). Marselan was the extract richer in catechins (132.99 ± 9.81 μg/g of dried matter), and it also contained a higher amount of phenolic compounds (49.08 ± 0.03 mg of gallic acid equivalent/g of dry matter and 10.02 ± 0.24 mg of proanthocyanidin content/g of dry matter). The antioxidant capacity of the extracts was assessed using three different colorimetric assays including 2,2-DiPhenyl-1-PicrylHydrazyl (DPPH), CUPRIC ion Reducing Antioxidant Capacity (CUPRAC), and Ferric Reducing Antioxidant Power (FRAP). As expected, Marselan exhibited the highest antioxidant activity; as well, the total phenolic and proanthocyanidin content were the highest. The stability of the Marselan extract incorporated into a commercial cream, was performed at three different temperatures (4, 25, and 50 °C), and four different concentrations (5, 4, 3, 2%), over a period of 4 months, using different methods such as centrifugation, Heat-Shock Cycles, pH, and viscosity. All Marselan hydro-glyceric extract formulations were proven to be stable over the entire 4 months, where the highest stability was achieved at 4 °C and the least at 50 °C. This study supports the suitability of the incorporation of phenolic extracts into commercial creams to enrich the cosmetic industry with effective, natural, and safe skincare products.

## 1. Introduction

During the winemaking process, which mostly consists of grape pressing and maceration, high amounts of organic wastes, mainly composed of grape pomaces (62%), lees (14%), stalks (12%) and dewatered sludge [1,2,3], are produced. These by-products are either used as compost, animal feed or discarded in landfills [4]. However, the low pH and the high amount of phenolic compounds, capable of resisting biological degradation, are one of the main environmental issues associated with these residues [5]. Indeed, grape pomaces have a high organic carbon content (31–54%), which is responsible for water pollution and foul odors, moreover pests and tannins, among other compounds, may affect the nearby flora and animals [6,7]. Different grape varieties are cultivated all over the world depending on the geographical distribution, climate changes, temperature trends, and the type of soil [8,9] and several studies confirmed that their content in bioactive molecules differs across varieties, especially between the red and white ones [10]. Among the different varieties, Marselan, Syrah, and Cabernet Franc are cultivated across vineyards worldwide [11], while Asswad Karech and Obeidi are Lebanese species. Obeidi in particular, is cultivated in the Beqaa Valley (Lebanon) and is a rare Lebanese grape variety. This variety is rich in valuable compounds and has a peculiar DNA profile, without links to other varieties, and is therefore classified as a purely Lebanese grape by Chateau Saint-Thomas. It is usually used for wine production and its valuable compounds, including fatty acids (13–19%), proteins (11%), carbohydrates (60–70%), and polyphenols (5–8%), remain in the winemaking by-products, especially in the seeds [12,13]. Polyphenols are the main secondary metabolites, including (a) non-flavonoid compounds such as benzoic acids (p-hydroxybenzoic acid, protocatechuic acid, and gallic acid) and cinnamic acids (coumaric acid, caffeic acid, ferulic acid, and chlorogenic acid), (b) flavonoids including flavan-3-ols (catechin, epicatechin), quercetin, flavanones and anthocyanins [14]. Different studies have been performed aiming at finding the best extraction method capable of effectively recovering these valuable compounds. If compared to conventional solid-liquid extraction, in which biomolecules diffuse through the cell membrane to reach the extraction solvent [15], the innovative extraction techniques damage cell walls/membranes and intensify polyphenol recovery. For example, infrared-assisted extraction efficiency is related to molecular bending, extending, and twisting [16,17]. On the other hand, high-voltage electrical discharges (HVED) cause an electrical breakdown in water, leading to an intense mixing, the production of shock waves, and bubble cavitation improving the efficiency of polyphenols’ extraction [18]. Their recovery was also enhanced by adding β-cyclodextrin (β-CD) to the extraction medium [19]. Despite the high efficiency of these innovative e methods, some of them still have cost and scalability challenges. Solid-liquid extraction using glycerol and water, on the other hand, can be considered a low-cost and effective way for the recovery of phenolic compounds, especially for cosmetic applications. The main advantage of this process is that the hydro-glyceric extractive solvent can be directly incorporated into many cosmetic formulations avoiding the subsequent step for solvent elimination. For this reason, and in line with green chemistry and circular economy, which aims at balancing economic growth, resource sustainability, and environmental protection, easy and low-cost extraction methods based on the use of natural and not toxic solvents, such as glycerin and water, are still preferred to ensure a safe and high-quality extract, easily scalable at industrial level, and, that can be exploited in different products [20].

These phenolic compounds have promising applications, especially in the cosmetic field and can be used instead of the synthetic ones to obtain effective, safe, and natural products, in line with the needs of modern society [21]. The main beneficial properties of polyphenols from grapes are related to their antioxidant, antifungal, and antimicrobial effects [22]. In light of this, they are currently used in cosmetics, especially in antiaging and skin lightening products, since they can improve skin hydration and smoothness, thus decreasing roughness, wrinkles depth, skin redness, and hyperpigmentation [23,24]. They can also protect the skin against oxidative stress and UV damage, thus acting as photoprotectors with a high sun protection factor [25]. Even more, they are capable of inhibiting the activity of proteinase, collagenase, and elastase, which are responsible for the degradation of collagen and elastin, thus ensuring skin firmness and elasticity [26]. Unfortunately, some of these compounds are highly unstable and have a low bioavailability in the skin, and their loading in specifically tailored nanocarriers is nowadays a challenging trend in the cosmetic industry [27]. These types of products are becoming more popular in this field since they promote payload efficacy and reduce the needed concentration [28].

The aim of this study was to evaluate and characterize the extracts obtained from different grape seed varieties and to design antioxidant and natural cosmetic formulations using the more effective extract obtained only using natural ingredients. For this reason, the seeds of five different grape varieties were separated from pomaces obtained after the wine-making process. The phytocomplexes were extracted using a simple and scalable method a mixture of water and glycerin as an extractive solution, since the latter is less erosive for the skin if compared with ethanol. The total phenolic and proanthocyanidin content and antioxidant activity have been measured. The extracts were formulated in natural-based creams, their stability was monitored by measuring pH, viscosity and their physicochemical composition after centrifugation and heat-shock cycles. The developed formulations may, therefore, serve as an economic booster to the local cosmetic market.

## 2. Materials and Methods

### 2.1. Plant Material

Fresh red pomaces of *Vitis vinifera* Marselan, Syrah and Obeidi were provided in September 2021 by Chateau Saint-Thomas, whereas Cabernet Franc and Asswad Karech were provided by Chateau Kefraya. Both wineries are located in the Beqaa Valley of Lebanon. The pomaces were then dehydrated in an airflow oven at 50 °C for 48 h. Seeds were then isolated from the skin using a vibrating multi sieve separator (ELE International, Loveland, CO, USA). Dried seeds were ground (Philips, Dubai, United Arab Emirates, MEA) and then sieved one more time to have a particle size ranging between 850 and 425 μm. Ground seeds were then packed in plastic bags and stored at room temperature and in the dark until further use.

### 2.2. Chemicals

All chemicals used in the experiments were of analytical grade. Folin-Ciocalteu reagent, sodium carbonate, gallic acid, 1,1-DiPhenyl-2- 113 Picryl Hydrazyl (DPPH), and 6-hydroxy-2,5,7,8-tetramethylchroman-2-carboxylic acid 114 (Trolox), were purchased from Sigma-Aldrich (Darmstadt, Germany). FRAP, CUPRAC and proanthocyanidin assay kits were provided from Bioquochem, (Asturias, Spain). Tween 20 (Polyoxyethylene-20) was purchased from Biotech, (Markham, ON, Canada).

All HPLC standards, gallic acid, protocatechuic acid, (+)-catechin, chlorogenic acid, caffeic acid, *p*-coumaric acid, rutin, D-malic acid, myricetin, *trans*-cinnamic, 3,5-dimethoxy-4-hydroxycinnamic acid, quercetin, luteolin and kaempferol were purchased from Sigma-Aldrich (Darmstadt, Germany). Water was purchased from LiChrosolv^®^ (Darmstadt, Germany), O-phosphoric acid 85% extra pure was provided by Scharlau, Turkey and acetonitrile was provided by VWR Prolabo^®^ Chemicals (France).

A commercial cream, mostly containing water, glycerin, stabilizers, and preservatives with no bioactive molecules, was locally purchased from a store. A commercial hydro-glyceric extract obtained from grape seed was kindly provided by a cosmetics society in France.

### 2.3. Extraction Process

The extraction of phenolic compounds from grape seeds was performed in a mixture of glycerin and water (50% *w/w*) at 40 °C for 2 h using a solid-to-liquid ratio (1:10 *w/w*). Briefly, 10 g of ground seeds were suspended in 100 g of the hydro-glyceric mixture, then maintained at 40 °C and under constant stirring during the extraction process. The obtained extract was then filtered through a glass wool and stored at 4 °C. Moreover, the commercial extract from grape seed, used as reference, also underwent the same extraction process using glycerin and water. The extraction process was performed in a digital water bath (JSR JSWB-22T, Gongju-city, Korea). To avoid solvent evaporation and the degradation or oxidation of phenolic compounds, all samples were closed and covered with aluminum foil during the extraction process.

### 2.4. Characterization of the Extract

#### 2.4.1. Total Phenolic Content

The Total Phenolic Content (TPC) was evaluated using the Folin-Ciocalteu assay [29]. Briefly, 200 µL of the extract solutions and 1000 µL of ten-fold diluted Folin-Ciocalteu reagent were added to 800 µL of sodium carbonate (75 g/L). The mixture was incubated at 60 °C for 10 min then cooled at 4 °C for 10 min. The absorbance was then measured at 750 nm using a UV192 Vis spectrophotometer (Biochrom Ltd., Cambridge, England). The pure solvent was used as blank control. A calibration curve was built using increasing concentrations of gallic acid and the Total Phenolic Content was expressed as milligrams of gallic acid equivalents (GAE) per gram of dry matter (mg GAE/g of dry matter).

#### 2.4.2. Proanthocyanidin Assay Kit

Proanthocyanidins were determined using a standard kit (Bioquochem, Asturias, Spain), based on the reaction of 4-dimethylaminocinnamaldehyde with the terminal units of the proanthocyanidin, which produces a green-blue colored compound. A total of 10 μL of the grape extract was added to 230 μL of reagent and 10 μL of 4-dimethylaminocinnamaldehyde were transferred in each well of 96-well clear-bottom plates. The plates were then stirred for 15 min and the absorbance was measured at 640 nm using a Thermo Scientific MULTISKAN GO reader. ProAnthoCyanidin (PAC) values were expressed as mg of ProAnthoCyanidin equivalents (PAC) per gram of dry matter (mg PAC/g of dry matter).

#### 2.4.3. Identification and Quantification of Polyphenols by High-Performance Liquid Chromatography (HPLC)

The identification and quantification of polyphenols contained in the extracts were performed using an HPLC autosampler (module 1260 Vialsampler) where the separation was obtained with a Kinetex*EVO C18 column (150 × 4.60 mm, 2.6 µm, 100 Å, Phenomenex, Casalecchio di Reno, Bologna, Italy). The mobile phase was composed of solution A, which was a mixture of 0.22 M phosphoric acid, and solution B, which was 100% acetonitrile, delivered at a flow rate of 0.8 mL/min. The volume of the sample injected was 10 μL. The gradient (*v*/*v*) was obtained by decreasing solvent A from 100% to 80% in 20 min, then to 70% in 35 min, up to 0% in 45 min remaining constant and stable up to 50 min. Finally, when the gradient reached 100%, it stayed stable for 5 min before the following injection [30]. Calibration curves were built using increasing concentrations of each standard (gallic acid, protocatechuic acid, (+)-catechin, chlorogenic acid, caffeic acid, *p*-coumaric acid, rutin, D-malic acid, myricetin, *trans*-cinnamic, 3,5-dimethoxy-4-hydroxycinnamic acid, quercetin, luteolin and kaempferol). For each curve, an equation was obtained and the R^2^ was calculated. All the analyses were performed on a wavelength of 280 nm.

All hydro-glyceric extracts were filtered before the injection through an Econofilter RC (0.45 μm, Ø 25 mm, Agilent Technologies, Milan, Italy) and set in the autosampler. All the analyses were performed in duplicate.

### 2.5. Antioxidant Activity

#### 2.5.1. DiPhenyl-2-PicrylHydrazyl Free Radical Scavenging Activity (DPPH)

Free radical scavenging activity was evaluated by measuring the ability of the phenolic compounds to reduce DPPH (2,2-DiPhenyl-PicrylHydrazyl) radical [31,32]. Briefly, 1450 μL of DPPH (0.06 mM) (Sigma Aldrich, St-Quentin Fallavier, France) was added to 50 μL of the grape extract or Trolox (positive control) (Sigma-Aldrich, St-Quentin Fallavier, France). After 30 min of incubation at room temperature in the dark, the absorbance was measured at 515 nm using pure methanol as a blank, and a calibration curve using Trolox as the standard was made. The antioxidant activity of the extracts was calculated according to the following formula:

$$Antioxidant\;activity\;\%=\frac{\left(absorbance\;of\;negative\;control-absorbance\;of\;sample\right)}{absorbance\;of\;negative\;control}\times 100$$

#### 2.5.2. Ferric Reducing Antioxidant Power Assay (FRAP)

The antioxidant activity was also evaluated by measuring the ability of the extracts to reduce the ferric complex at an acidic pH (FRAP, Bioquochem, Asturias, Spain). Briefly, 10 μL of the diluted extract, or standard, was added to 220 μL of the ready-to-use FRAP working solution. The absorbance was measured using a plate reader at 593 nm after 4 min of gentle stirring. A standard curve was built to measure the absorbance as a function of iron II (Bioquochem, Asturias, Spain) concentrations. The antioxidant activity was expressed as mM of Iron (II) equivalents.

#### 2.5.3. CUPRIC Ion Reducing Antioxidant Capacity Assay (CUPRAC)

The total antioxidant capacity of extracts was also measured using the CUPRAC assay kit (Bioquochem, Asturias, Spain), based on the oxidation of the copper (II)-neocuproine (2,9-dimethyl-1,10-phenanthroline). A total of 40 μL of the diluted extract, or standard, was added to 200 μL of the previously prepared working solution. The mixture was incubated at room temperature for 30 min, and the absorbance was measured at 450 nm using a plate reader. Results were expressed as mM of Trolox equivalents (TE mM).

### 2.6. Incorporation of the Extracts in Cosmetic Creams and Stability Tests

The hydro-glyceric extract from the Marselan variety was incorporated at different percentages (2%, 3%, 4%, 5%) in a commercial cream, purchased in a local pharmacy. The cream without extract (0%) was used as a negative control. The commercial hydro-glyceric extract was incorporated (5%) in the commercial cream as well, and used as reference because it was obtained using the same extraction solvent (50% *w/w*) glycerin and water at the same temperature of 40 °C for 2 h. However, the variety of grape used to obtain the commercial extract was not specified by the supplier.

### 2.7. Stability Tests

Three batches of creams were prepared containing 6 creams each (5%, 4%, 3%, 2%, 0% of the Marselan hydro-glyceric extract, and 5% of a commercially purchased extract) stored at 3 different temperatures (4, 25, 50 °C) for the following stability tests:

#### 2.7.1. Centrifuge Test

The stability of the creams was evaluated by a centrifugation method using a Heraeus Primo R Centrifuge (Thermo Scientific™ Heraeus™, Hanau, Germany) at 3000 rpm for 30 min at room temperature to observe possible instabilities (phase separation, precipitation, coalescence, and sedimentation) [33].

#### 2.7.2. Heat-Shock Cycles

Physicochemical variations were checked after heating and cooling cycles. All creams were stored at 4 °C for 24 h and were directly removed and placed at 50 °C for another 24 h. This procedure was repeated for 4 cycles [34].

#### 2.7.3. Viscosity and pH Tests

The appearance of the 3 batches of creams stored at three different temperatures (4, 25, and 50 °C) was optically observed. Viscosity values of the cream at room temperature (25 °C) were measured by using a Brookfield DV-II + pro viscometer (Brookfield Engineering Laboratories Inc., USA) fitted with an S64 spindle at 100 rpm. The pH values of the samples were determined at room temperature (25 °C) using a pH meter (WTW, Weilheim, Germany). All measurements were taken over four months.

### 2.8. Total Phenolic Content and Antioxidant Activity of the Creams

The creams (with or without the extract) were first diluted with a mixture of polysorbate 20 and water (1:5 *v*/*v*) in a ratio of 1:3 (*v*/*v*). After dilution, the dispersions were maintained under stirring for 15 min at 400 rpm, at a temperature of 50 °C [35]. The stock solutions of the 3 batches of creams, were used for the Total Phenolic Content and Antioxidant Activity analysis. All the measurements were taken over four months.

### 2.9. Statistical Analysis

The means and error bars were represented in all the figures. Variance analysis (ONE-WAY ANOVA) and Least Significant Difference test (LSD) were evaluated by STATGRAPHICS^®^ Centurion XVI (StatPoint Technologies, Inc.). In bar diagrams, the means sharing the same letter are not significantly different from each other (with a *p* > 0.05). All analyses were repeated at least 3 times.

## 3. Results and Discussion

### 3.1. Extraction and Quantification of Phenolic Content

#### 3.1.1. Extraction

**Figure 1:**
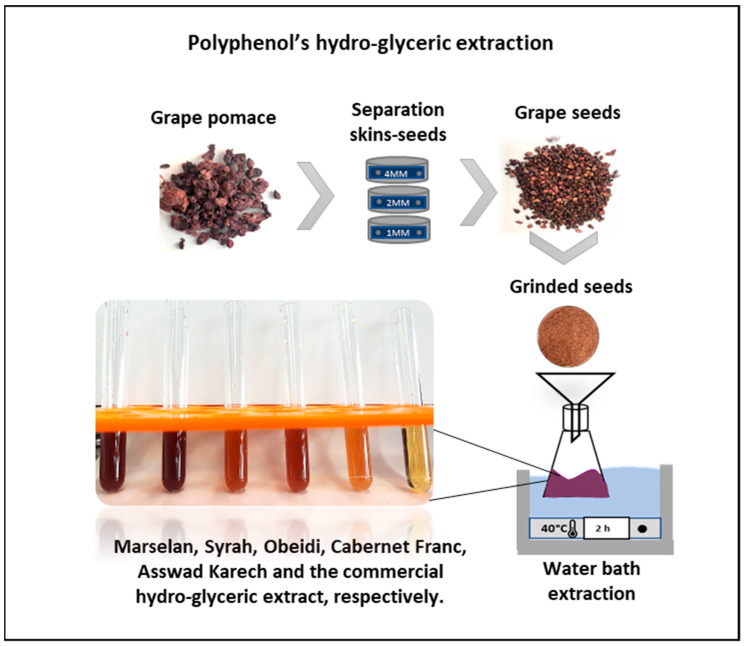
Schematic representation of the extraction process performed using grape seeds separated from the pomaces of Marselan, Syrah, Obeidi, Cabernet Franc, and Asswad Karech.

The extracts were obtained by maceration of the ground seeds (10 g) in 100 g of a mixture of glycerin and water (50% *w*/*w*) at 40 °C for 2 h (Figure 1). The use of toxic solvents was avoided, and glycerin was used instead of ethanol, since it is safer and also a skin emollient solvent, which prevents the skin from dryness [36]. Ferreira et al. used ethanol as medium for the extraction of polyphenols, but this water-soluble solvent is also considered a more erosive compound when applied to the skin compared to glycerin, which is more eco-friendly as well [37]. The same extraction procedure was applied to the recovery of the main bioactive compounds from the five different varieties of grape seeds: Marselan, Syrah, Obeidi, Cabernet Franc, and Asswad Karech.

#### 3.1.2. Total Phenolic Content and ProAnthoCyanidin Content of the Hydro-Glyceric Extracts

**Figure 2:**
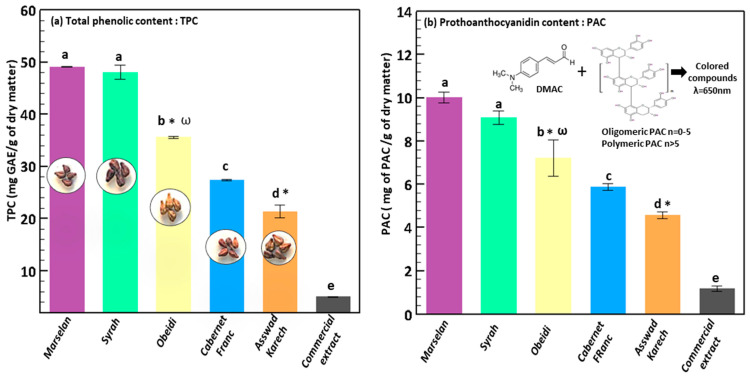
Total phenolic content (**a**, panel on the left), proanthocyanidin content (**b**, panel on the right) of the hydro-glyceric extracts obtained from the grape seeds of Marselan, Syrah, Obeidi, Cabernet Franc and Asswad Karech, respectively. Mean values ± standard deviations were reported. Each letter indicates the same value (*p* > 0.05), symbol ω indicates a white variety, and symbol * indicates Lebanese variety.

The total phenolic and the proanthocyanidin content of the extracts obtained from the seeds of the international red grape varieties (Marselan, Syrah, and Cabernet Franc) and from Lebanese varieties (white Obeidi and red Asswad Karech) were measured (Figure 2a,b, left and right panel). The total phenolic content of the extracts obtained from the Marselan and Syrah varieties was the highest, ~49.1 mg GAE/g of dry matter, without differences between the two varieties (*p* > 0.05). That of the other extracts was lower compared to that of Marselan and Syrah (*p* < 0.05): 35.58 ± 0.20 mg GAE/g of dry matter from Obeidi, 27.36 ± 0.10 mg GAE/g of dry matter from Cabernet Franc and 21.36 ± 1.18 mg GAE/g of dry matter from Asswad Karech. Similar results were observed, after alcoholic fermentation, by Ju. Y et al., which found that Marselan and Syrah have the highest total phenolic content among the studied seeds from different grape varieties [38]. Moreover, the extract from the Obeidi variety had a total phenolic content that was only 30% lower than that of the extracts obtained from the international varieties (Marselan and Syrah) and higher than that of the others, suggesting that Obeidi seeds are a valuable source of polyphenols. On the contrary, the extract of the other Lebanese variety (Asswad Karech), had the lower total phenolic content among all the others (Figure 2a). The lowest total phenolic content was found for the commercial extract (5.00 ± 0.12 mg GAE/g of DM), although the variety used to obtain this extract was not specified by the supplier. The measured values of the polyphenols found by Sandhu et al. and extracted using a mixture of acetone, water, and acetic acid, were in the same range [39]. To note that, the total phenolic content of Asswad Karech (21.36 ± 1.18 mg GAE/g of dry matter) was the lowest among the studied varieties but was higher than that of the commercial extract. The proanthocyanidin content disclosed a similar trend. Indeed, the extracts from Marselan and Syrah had the highest values (~9.5 mg proanthocyanidin content/g of dry matter), with no significant differences between the samples (*p* > 0.05), while those of the other varieties were lower. In particular, that of the extract from Obeidi was 7.22 ± 0.84 mg proanthocyanidin content/g of dry matter followed by that of Cabernet Franc, 5.87 ± 0.15 mg proanthocyanidin content/g of dry matter, and Asswad Karech, 4.57 ± 0.15 mg proanthocyanidin content/g of dry matter. Like the total phenolic content, the proanthocyanidin content of the extract obtained from the Lebanese variety, Obeidi, was only 30% lower in comparison with that of the international varieties (Marselan and Syrah). In all the obtained extracts, there was a direct correlation between the total phenolic content and Proanthocyanidin content confirming the simultaneous presence of all the secondary metabolites in the grape seeds. These results suggested that the extraction using a mixture of water and glycerol allowed to obtain an extract effectively rich in bioactive molecules and the percentage of amelioration of the tested extracts was higher than that of the commercial one. Indeed, that of extract from Marselan was 882%, that of extract from Obeidi was 862%, that of extract from Cabernet Franc was 611% and that of extract from Asswad Karech was 446%. All were better than that of the commercial one, 327%.

#### 3.1.3. Identification and Quantification by HPLC

**Figure 3:**
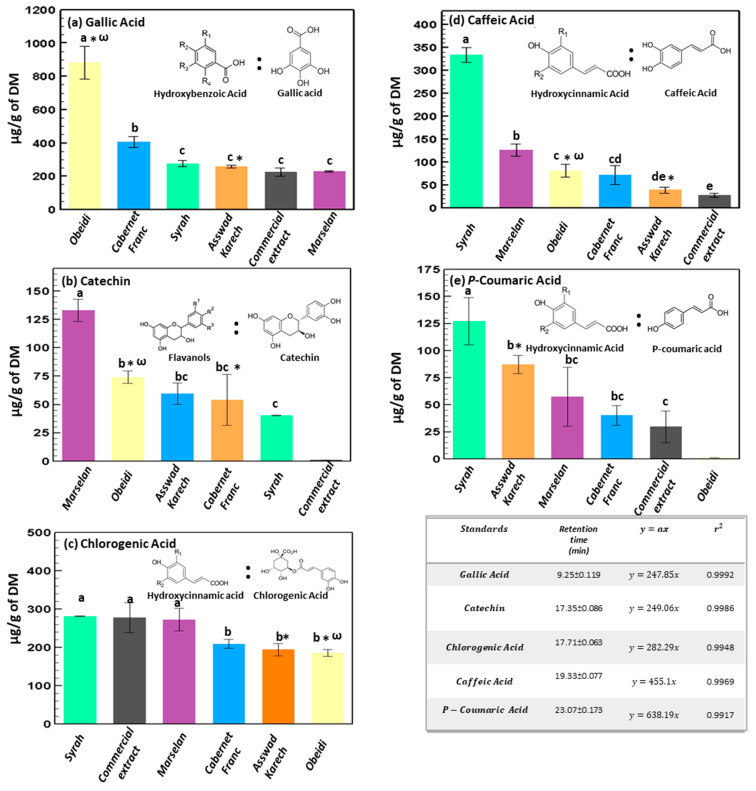
Quantification (μg/g of dry matter, DM) of the main polyphenols: separated and identified by HPLC in the hydro-glyceric extracts obtained from the grape seeds of Marselan, Syrah, Obeidi, Cabernet Franc and Asswad Karech. Mean values ± standard deviations were reported. Each letter indicates the same value (*p* > 0.05), symbol ω indicates a white variety, and symbol * indicates Lebanese variety.

Phenolic compounds (anthocyanins, proanthocyanins, hydrolyzable tannins, flavonols, flavan-3-ols, flavanones, flavones, and phenolic acids) contained in the different extracts were detected and quantified by HPLC (Supplementary Material, Figure S1). Five different types of polyphenols were detected and quantified (Figure 3a–e). The main compounds were gallic acid, catechins, chlorogenic acid, caffeic acid, and *p*-coumaric acid, according to Vidal-Casnella et al. [40]. Gallic acid, a member of the hydroxybenzoic acids family, is well known for its antioxidant, antiapoptotic, cardioprotective, neuroprotective, antiproliferative, and anticancer properties [41,42]. It was the most abundant in the extract from Obeidi (883.05 ± 98.59 μg/g of dry matter), while in the other extracts, the amount of gallic acid was significantly lower as a function of the grape variety tested. It decreased as follows: Cabernet Franc 407.11 ± 31.66 μg of gallic acid/g of dry matter; Syrah 276.25 ± 18.63 μg/g of dry matter; Asswad Karech 257.33 ± 7.96 μg/g of dry matter, the commercial extract 224.26 ± 24.81 μg/g of dry matter and Marselan 231.22 ± 4.89 μg/g of dry matter (Figure 3a). These results, especially those of the extract from Obeidi grape seeds, are in accordance with Mandic et al., which utilized as extraction solvent, ethanol instead of water and glycerin [43]. Moreover, the amount of gallic acid found in the Obeidi extract was significantly higher than that found in the other extracts (*p* < 0.05), while no significant differences in its concentration were detected between the other varieties (*p* > 0.05).

Catechins, from the family of flavanols, were also detected in the different extracts (Figure 3b). These polyphenols are mostly present in tea, grapes, apples, and berries and have almost the same health benefits as gallic acid. Additionally, they have good anti-diabetic, anti-obesity, anti-infectious and hepatoprotective properties as well [44]. The highest content of catechins was found in Marselan extract (132.99 ± 9.81 μg/g of dry matter), followed by Obeidi (74.10 ± 5.40 μg/g of dry matter), Asswad Karech (59.66 ± 9.32 μg/g of dry matter), Cabernet Franc (54.14 ± 22.45 μg/g of dry matter) and Syrah (40.39 ± 0.23 μg/g of dry matter). On the contrary, the amount of catechins found in the commercial extract was negligible. No significant differences were detected between the concentrations of catechins in the hydro-glyceric extracts from Asswad Karech, Cabernet Franc, and Obeidi (*p* > 0.05).

Ferulic, caffeic, *p*-coumaric, and chlorogenic acids are classified as hydroxycinnamic acids, and some of them were detected in the extracts from grape seeds. Chlorogenic acid is a strong antioxidant with wound healing effects, due to its capability of boosting collagen synthesis [45]. Caffeic and coumaric acids have preventive effects against chronic diseases and cancer, related to their bioavailability in the human body, where *p*-coumaric acid has been found to be higher than chlorogenic acid, when unbounded to proteins [46]. Chlorogenic acid was the most abundant in Syrah and Marselan, ~274 μg/g of dry matter, and in the commercial extract (~272 μg/g of dry matter), with no significant differences among them (*p* > 0.05) (Figure 3c). The lowest concentration of chlorogenic acid was detected in the extracts from Cabernet Franc, Asswad Karech, and Obeidi with ~196 μg/g of dry matter (*p* > 0.05). Similar results were found by Stanclu, G. et al., which analyzed the extracts from Cabernet Franc and Asswad Karech varieties [47]. The highest amount of caffeic, 334.17 ± 16.57 μg/g of dry matter, and *p*-coumaric acids, 127.06 ± 21.78 μg/g of dry matter, were found in an extract from Syrah (Figure 3d,e). Whereas, in the other extracts, the amount of these two compounds was significantly lower. The content of caffeic acid was higher in the extract from Marselan (126.88 ± 13.20 μg/g of μg/g of dry matter), followed by that from Obeidi and Cabernet Franc (~76.45 ± 14.27 μg/g of μg/g of dry matter) with no significant difference (*p* > 0.05) between the two values. The lowest amount was detected in extracts from Asswad Karech and Commercial, ~34 μg/g of μg/g of dry matter. The amount of *p*-coumaric acid detected in the extract from Asswad Karech was the highest (87.26 ± 2.49 μg/g of μg/g of dry matter), followed by that from Marselan and Cabernet Franc (~49 μg/g of μg/g of dry matter) and that of the commercial one (29.76 ± 14.57 μg/g of μg/g of dry matter). On the other hand, this compound was not detected in Obeidi extract. According to the previous results, the quantification of the different phenolic compounds in each extract confirmed the presence of several polyphenols, which, therefore, positively affects the antioxidant activity of the different grape varieties.

### 3.2. Antioxidant Activity of the Extract by DPPH, FRAP, and CUPRAC Assays

**Figure 4:**
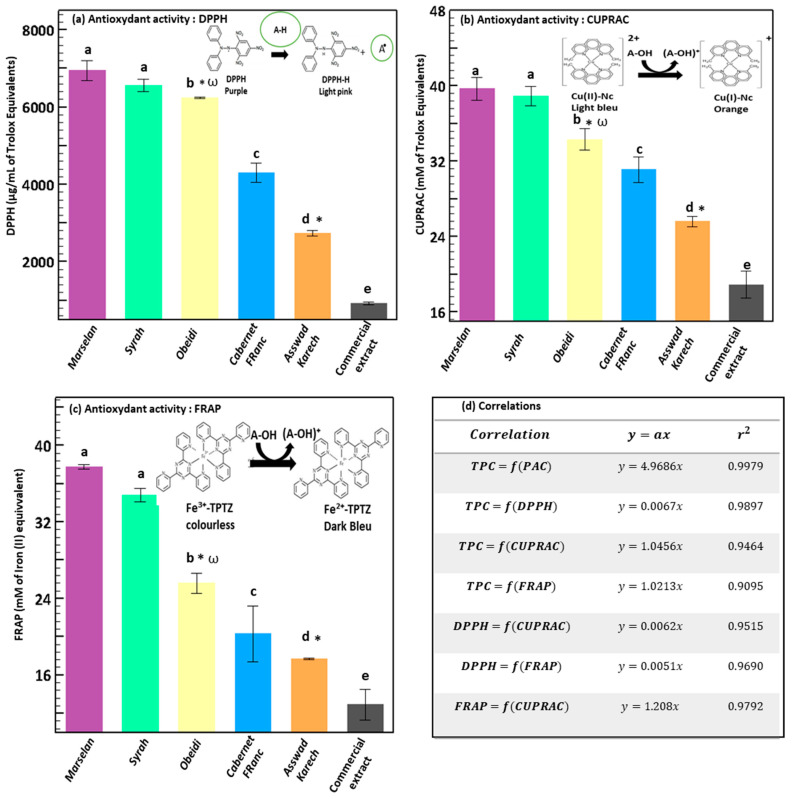
Antioxidant activity of the hydro-glyceric extracts obtained from the grape seeds of Marselan, Syrah, Obeidi, Cabernet Franc, and Asswad Karech, measured by means of DPPH (**a**), CUPRAC (**b**), and FRAP (**c**) colorimetric assays. (**d**) Mean values ± standard deviations were reported. Each letter indicates the same value (*p* > 0.05), symbol ω indicates a white variety, and symbol * indicates Lebanese variety.

The antioxidant activity of the obtained extracts was evaluated by measuring their free-radical scavenging activity, using three different assays: DPPH (Figure 4a), CUPRAC (Figure 4b), and FRAP (Figure 4c). The extracts from Marselan and Syrah had the highest antioxidant activity: ~6743 µg/mL of trolox equivalents (*p* > 0.05) by the DPPH assay (Figure 4a), ~39 mM of trolox equivalents (*p* > 0.05) by the CUPRAC assay (Figure 4b) and ~36 mM of Iron equivalents (*p* > 0.05) by the FRAP test (Figure 4c). The antioxidant activities of the extracts from Obeidi, Cabernet Franc and Asswad Karech, measured with DPPH assay were 6235 ± 18, 4302 ± 242, 2731 ± 69 µg/mL of trolox equivalents, respectively (Figure 4a). The same trend was found in measuring the antioxidant activity with the CUPRAC and FRAP assays. Indeed, among the three extracts, that from Obeidi had 34 ± 2 mM of trolox equivalents and 26 ± 1 mM of Iron (II) equivalents, followed by that from Cabernet Franc (30 ± 1 mM of trolox equivalents and 18 ± 0.07 mM of Iron equivalents) and that from Asswad Karech (26 ± 1 mM of trolox equivalents and 13 ± 2 mM of Iron equivalents) (Figure 4b,c). The antioxidant activity values of the commercial extract were the lowest: 921 ± 41 µg/mL of Trolox equivalents measured with DPPH, 19 ± 2 mM of trolox equivalents measured with CUPRAC, and 13 ± 2 mM of Iron equivalents measured with FRAP. These results are in agreement with those found by Xia, E-Q. et al., in particular with those obtained using the DPPH colorimetric assay [48]. According to these findings, increased antioxidant activity and inhibition of free radicals were obtained using the extracts with the highest phenolic content (Figure 4d). As expected, the extract obtained from Marselan grape seeds had the highest antioxidant activity, probably due to the high polyphenols and proanthocyanidin content. Based on this hypothesis, Soobratteea et al. also found that the most antioxidant compounds were procyanidin dimers, thus explaining their effects through the Marselan variety since it derives from the anthocyanidins family [49]. Moreover, as previously reported by Yilmaz et al. and Zhou et al., catechins, being the major phenolic compounds present in grape seeds, have been found to exert the strongest radical scavenging activity among the majority of polyphenols. Therefore, since the extract from Marselan was the richest in catechins (Figure 3b), its strong antioxidant activity could be related to the presence of a wide amount of these compounds [1,50]. Given that, and considering the promising beneficial properties of this variety, seed extract from Marselan has been chosen to be incorporated into a commercial cream.

### 3.3. Stability Tests of Creams Containing Hydro-Glyceric Extract

#### 3.3.1. Centrifuge Test and Heat-Cool Cycles

**Figure 5:**
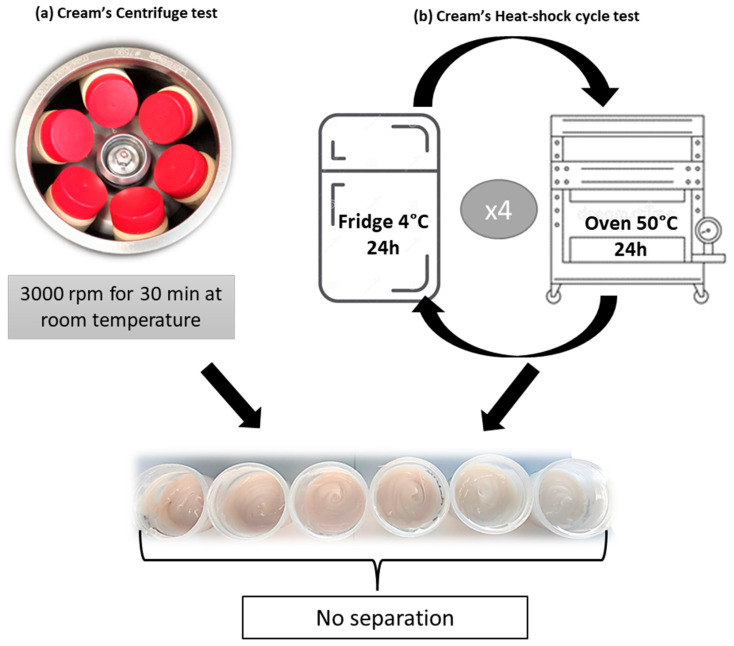
Schematic representation of the tests performed on creams containing 0, 2, 3, 4, 5%, Marselan extract, or 5% of the commercial extract: centrifuge test (**a**), heat-shock cycle (**b**) repeated four times.

The stability of the creams containing the Marselan extract against external factors was evaluated via a centrifugation test, which allowed the assessment of their stability as a function of the centrifugal force. To perform the tests, a batch of six creams containing increasing concentrations of Marselan extract (2, 3, 4, 5%) or 5% of commercial extract (positive control) or without the extract (0 %, negative control) was prepared. Each sample was placed in centrifugal tubes and centrifuged at 3000 rpm for 30 min at room temperature (25 °C) (Figure 5a).

The stability was, furthermore, tested upon the effect of heat-shock or freeze-thaw cycles. This technique has been used to observe the changes in physicochemical properties of the creams (i.e., separation and difference in color due to temperature variations). Four cycles between refrigerator (4 °C) and oven (50 °C) were performed every 24 h (Figure 5b). No phase separation (i.e., coalescence or sedimentation) or color change, was observed after centrifuge and heat-shock treatments. Ferreira, S. M. et al. also confirmed the stability of the creams containing polyphenols over 2 months, as no separation was detected after centrifugation or thermal stress [37]. From a rheological point of view, polyphenols were found to be stable when incorporated into commercial creams and would, therefore, serve as a great pioneer for the skincare industry.

#### 3.3.2. Measurements of pH and Viscosity

**Figure 6:**
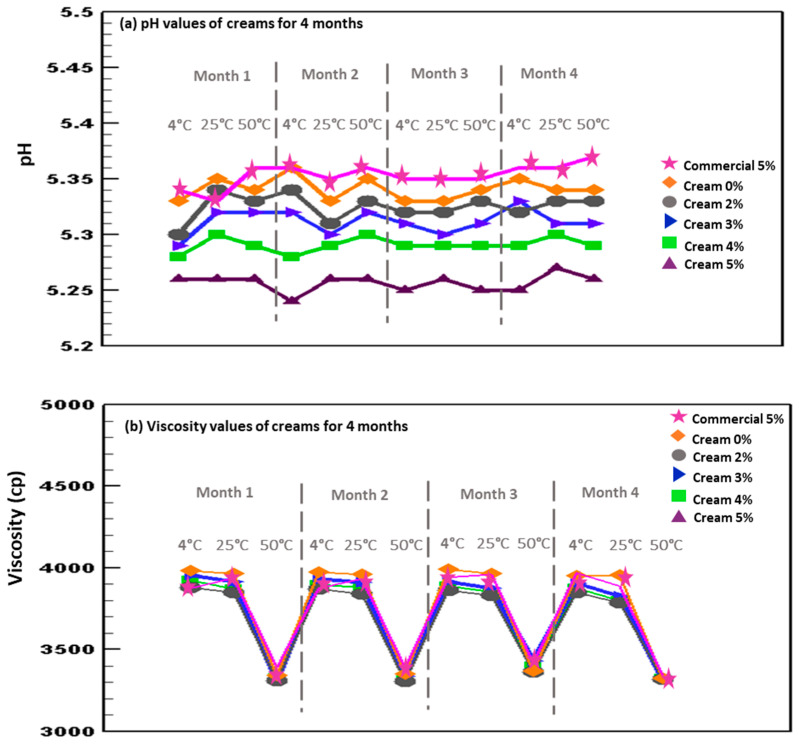
Values of pH (**a**, upper panel) and viscosity (**b**, lower panel) of creams, containing 0, 2, 3, 4, 5%, Marselan extract, or 5% of the commercial extract, and stored for 4 months at 4, 25 and 50 °C.

The pH and viscosity of the creams were also measured at scheduled times, storing the samples at 4, 25, and 50 °C. The changes in these parameters are predictive of the samples’ stability as well [51]. Viscosity is the measure of the ability of formulations to resist deformation or, inversely, the measure of their capability of flowing as a function of the sheer stress and the temperature applied. From the practical point of view, it allows to predict the spread-ability of the topical formulations on the skin. The viscosity of the creams containing the extracts was tested over a period of 4 months at three different temperatures: 4 °C (fridge), 25 °C (room temperature), and 50 °C (oven) (Figure 6b, lower panel). The viscosity varied as a function of the temperature. Indeed, it reached the highest value at 4 °C, and progressively decreased to the lowest value at 50 °C. Similarly, Ishaq M. A. et al., also observed that the viscosity of the cream changes as a function of the temperature gradient, as it decreased at higher temperatures (~40 °C) and increased at lower temperatures (~8 °C) [52]. The measured viscosities of the creams were ~3990 cP at 4 °C, ~3825 cP at 25 °C, and ~3375 cP at 50 °C (Figure 6b, lower panel), irrespective of the used extract and concentration (*p* > 0.05).

The pH of the samples was also determined at different storage temperatures over a period of 4 months: 4 °C (fridge), 25 °C (room temperature), and 50 °C (oven) (Figure 6a, upper panel). The pH of the creams containing the extracts (Marselan or the commercial one) maintained their stability (~5.3) during the four months of evaluation and did not change as a function of both the used extract and the concentration (*p* > 0.05 between time, type of extract and concentrations). Similar results have been previously found by Rafique, M. et al., as they also found pH values within the range of 4.8 and 5.4 over a period of 3 months [53]. Considering that the pH of the skin ranges between 4.5 and 6.0, the creams containing grape seed extract seem to be suitable for skin application, as previously reported by Rafique, M. et al., [54]. Overall results suggested the potential of these formulations as effective skincare products, easily marketable by cosmetic industries.

### 3.4. Total Phenolic Content and Antioxidant Activity of the Incorporated Extract

**Figure 7:**
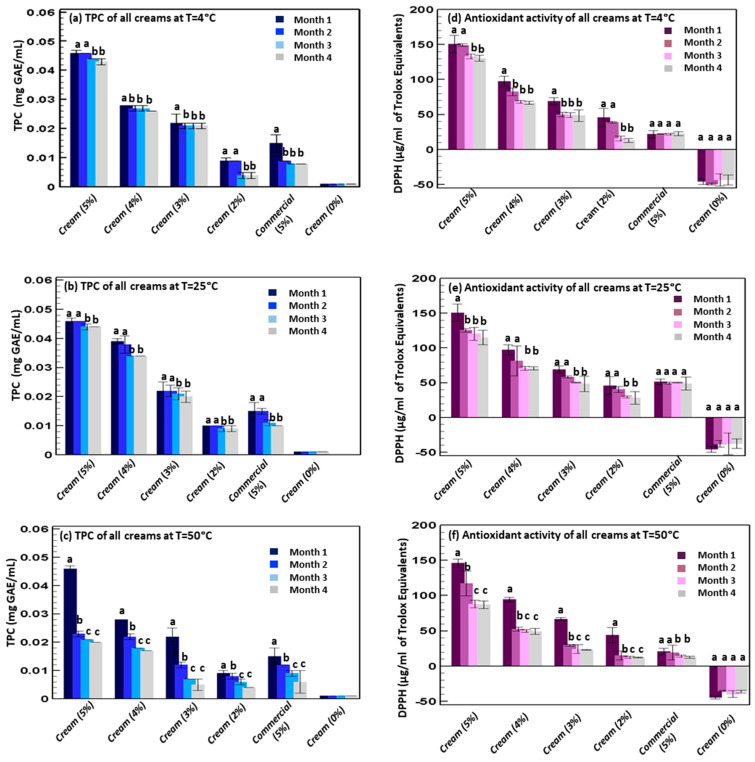
Total Phenolic Content (TPC) measured at 4 °C (**a**), 25 °C (**b**) and 50 °C (**c**) and antioxidant activity (DPPH assay) measured at 4 °C (**d**), 25 °C (**e**) and 50 °C (**f**) of the creams containing 0, 2, 3, 4, 5% of Marselan hydro-glyceric extract, or 5% of the commercial extract. Mean values ± standard deviations were reported. Each letter indicates the same value (*p* > 0.05).

The obtained results confirmed that the incorporation of the extract into the cream did not modify physico-chemical stability and properties of the final product. Finally, their biological properties including the total phenolic content (by Folin-Ciocalteu assay) and the trolox equivalents (by DPPH scavenging activity) were evaluated in vitro as a function of time, at 4, 25, and 50 °C (Figure 7). At 4 and 25 °C, the cream containing 5% of the Marselan extract had the highest Total Phenolic Content (0.046 ± 0.001 mg GAE/mL) and antioxidant activity (150 ± 13 µg/mL of trolox equivalents), and the values remained constant during the first 2 months without significant differences (*p* > 0.05 among values at 4 or 25 °C, at 1 or 2 months), except for the trolox equivalents at 25 °C, which decreased. During the 3rd and 4th months, at 4 °C, the Total Phenolic Content slightly decreased to 0.043 ± 0.001 mg GAE/mL (*p* < 0.05 compared to the values at 1 and 2 months), while the antioxidant activity significantly decreased up to 130 ± 4 µg/mL of trolox equivalents (*p* < 0.05 compared to the values at 1 and 2 months). At 25 °C, the Total Phenolic Content decreased up to 0.044 ± 0.001 mg GAE/mL (*p* < 0.05 compared to the values at 1 and 2 months and *p* > 0.05 for the same months at 4 °C), during the 3rd and 4th months, while the antioxidant activity significantly decreased during the 2nd month up to 115 ± 10 µg/mL of trolox equivalents (*p* < 0.05 compared to the values at 1 month) and remained constant up to the 4th month. Otherwise, at 50 °C the cream containing 5% of the Marselan extract had the highest phenolic content of 0.046 ± 0.001 mg GAE/mL and an antioxidant activity of 146 ± 6 µg/mL of trolox equivalents, which severely decreased up to 0.020 ± 0.001 mg GAE/mL and 87 ± 6 µg/mL of trolox equivalents during the 3rd month and remained constant up to the 4th month (*p* < 0.05), according to the previous results [55].

Clearly, when using lower concentrations of extract (2, 3, 4%), the Total Phenolic Content and antioxidant activity were lower, at 4 °C, where the Total Phenolic Content was 0.028 ± 0.001 mg of GAE/mL for the 4%, 0.022 ± 0.003 mg of GAE/mL for the 3% and 0.009 ± 0.001 mg of GAE/mL for the 2%. The values remained stable for the upcoming 3 months. The same trend was detected for the antioxidant activity, as it was 98 ± 8 µg/mL of trolox equivalents for the 4%, 66 ± 5 µg/mL of trolox equivalents for the 3%, and 46 ± 13 µg/mL of trolox equivalents for the 2%, during the 1st month. From the 2nd to the 4th months, these values decreased up to 67 ± 2, 48 ± 8 and 13 ± 3 µg/mL of trolox equivalents. At 25 and 50 °C, the behavior was similar, and the creams were stable for up to 2 months after which the values decreased. Vural, N. et al., found similar results, even though they used ethanol as an extraction solvent [56].

As expected, the negative control (cream without extract) had the lowest values of Total Phenolic Content and antioxidant activity since its components do not have any antioxidant power. The positive control (cream with commercial extract) had values that were lower than those of the cream containing the 5% of Marselan extract and similar to those of the cream containing 2% of the Marselan extract. Indeed, at 4 °C, the Total Phenolic Content was 0.015 ± 0.003 mg GAE/mL and antioxidant value was 23 ± 2.8 µg/mL of trolox equivalents. The values decreased after the first month, with any statistical differences among the 3 following months (*p* > 0.05). At 25 and 50 °C, the values decreased during the third and fourth months (*p* < 0.05).

Overall results confirmed that the cream containing 5% of Marselan extract had the highest phenolic content and antioxidant activity and all the creams were more stable at 4 °C and less stable at 50 °C, irrespective of the concentration of extract. Furthermore, all the stability tests including pH, Viscosity, Total Phenolic Content, and antioxidant activity, were extended for more than 8 months, and it was proven that all creams remained stable.

## 4. Conclusions

The performed studies confirmed that the maceration of grape seed pomace in a hydro-glyceric mixture is an easy and environmentally-friendly method to obtain a promising extract. The extract obtained from the Marselan variety had the highest phenolic content and antioxidant activity followed by that from Syrah, Obeidi, Cabernet Franc, and Asswad Karech. Moreover, the extract from the Lebanese variety, Obeidi, has proven to have a significant antioxidant potential when compared to the international ones. Additionally, the Marselan extract can be effectively added to a commercial cream, at increasing concentrations of up to 5%, without affecting its physico-chemical properties and stability. The creams containing the Marselan extract were stable when stored for a period of 4 months and had strong antioxidant activity, thus supporting the development of commercial formulations specifically designed for skincare.

## Data Availability

Data is contained within the article.

## Supplementary Materials

The following are available online at https://www.mdpi.com/article/10.3390/antiox11071348/s1, Figure S1: Representative chromatogram of the performed HPLC analysis.

## References

[1] Yilmaz Y., Toledo R.T. (2004). Major Flavonoids in Grape Seeds and Skins: Antioxidant Capacity of Catechin, Epicatechin, and Gallic Acid. J. Agric. Food Chem..

[2] Yu J., Ahmedna M. (2013). Functional Components of Grape Pomace: Their Composition, Biological Properties and Potential Applications. Int. J. Food Sci. Technol..

[3] Ruggieri L., Cadena E., Martínez-Blanco J., Gasol C.M., Rieradevall J., Gabarrell X., Gea T., Sort X., Sánchez A. (2009). Recovery of Organic Wastes in the Spanish Wine Industry. Technical, Economic and Environmental Analyses of the Composting Process. J. Clean. Prod..

[4] Barbanera M., Cardarelli A., Carota E., Castellini M., Giannoni T., Ubertini S. (2021). Valorization of Winery and Distillery By-Products by Hydrothermal Carbonization. Sci. Rep..

[5] Bustamante M.A., Moral R., Paredes C., Pérez-Espinosa A., Moreno-Caselles J., Pérez-Murcia M.D. (2008). Agrochemical Characterisation of the Solid By-Products and Residues from the Winery and Distillery Industry. Waste Manag..

[6] Ibn Ferjani A., Jellali S., Akrout H., Limousy L., Hamdi H., Thevenin N., Jeguirim M. (2020). Nutrient Retention and Release from Raw Exhausted Grape Marc Biochars and an Amended Agricultural Soil: Static and Dynamic Investigation. Environ. Technol. Innov..

[7] Mora-Garrido A.B., Cejudo-Bastante M.J., Heredia F.J., Escudero-Gilete M.L. (2022). Revalorization of Residues from the Industrial Exhaustion of Grape By-Products. LWT.

[8] Biasi R., Brunori E., Ferrara C., Salvati L. (2019). Assessing Impacts of Climate Change on Phenology and Quality Traits of *Vitis Vinifera* L.: The Contribution of Local Knowledge. Plants.

[9] Santos J.A., Fraga H., Malheiro A.C., Moutinho-Pereira J., Dinis L.-T., Correia C., Moriondo M., Leolini L., Dibari C., Costafreda-Aumedes S. (2020). A Review of the Potential Climate Change Impacts and Adaptation Options for European Viticulture. Appl. Sci..

[10] Lan Y., Liu M., Zhang X., Li S., Shi Y., Duan C. (2022). Regional Variation of Chemical Characteristics in Young Marselan (*Vitis Vinifera* L.) Red Wines from Five Regions of China. Foods.

[11] Li M., Guo Z., Jia N., Yuan J., Han B., Yin Y., Sun Y., Liu C., Zhao S. (2019). Evaluation of Eight Rootstocks on the Growth and Berry Quality of ‘Marselan’ Grapevines. Sci. Hortic..

[12] Antonic B., Dordevic Jancikova S., Dordević D., Tremlová B. (2020). Grape Pomace Valorization: A Systematic Review and Meta-Analysis. Foods.

[13] Shi J., Yu J., Pohorly J.E., Kakuda Y. (2003). Polyphenolics in Grape Seeds-Biochemistry and Functionality. J. Med. Food.

[14] Fraga C.G., Croft K.D., Kennedy D.O., Tomás-Barberán F.A. (2019). The Effects of Polyphenols and Other Bioactives on Human Health. Food Funct..

[15] Maroun R.G., Rajha H.N., Vorobiev E., Louka N., Galanakis C.M. (2017). 7-Emerging Technologies for the Recovery of Valuable Compounds From Grape Processing By-Products. Handbook of Grape Processing By-Products.

[16] Abi-Khattar A.-M., Rajha H.N., Abdel-Massih R.M., Maroun R.G., Louka N., Debs E. (2019). Intensification of Polyphenol Extraction from Olive Leaves Using Ired-Irrad®, an Environmentally-Friendly Innovative Technology. Antioxidants.

[17] Cheaib D., El Darra N., Rajha H.N., El-Ghazzawi I., Mouneimne Y., Jammoul A., Maroun R.G., Louka N. (2018). Study of the Selectivity and Bioactivity of Polyphenols Using Infrared Assisted Extraction from Apricot Pomace Compared to Conventional Methods. Antioxidants.

[18] Rajha H.N., Boussetta N., Louka N., Maroun R.G., Vorobiev E. (2015). Electrical, Mechanical, and Chemical Effects of High-Voltage Electrical Discharges on the Polyphenol Extraction from Vine Shoots. Innov. Food Sci. Emerg. Technol..

[19] Rajha H.N., Chacar S., Afif C., Vorobiev E., Louka N., Maroun R.G. (2015). β-Cyclodextrin-Assisted Extraction of Polyphenols from Vine Shoot Cultivars. J. Agric. Food Chem..

[20] Pagano I., Campone L., Celano R., Piccinelli A.L., Rastrelli L. (2021). Green Non-Conventional Techniques for the Extraction of Polyphenols from Agricultural Food by-Products: A Review. J. Chromatogr. A.

[21] Hoss I., Rajha H.N., El Khoury R., Youssef S., Manca M.L., Manconi M., Louka N., Maroun R.G. (2021). Valorization of Wine-Making By-Products’ Extracts in Cosmetics. Cosmetics.

[22] Baroi A.M., Popitiu M., Fierascu I., Sărdărescu I.-D., Fierascu R.C. (2022). Grapevine Wastes: A Rich Source of Antioxidants and Other Biologically Active Compounds. Antioxidants.

[23] Kanlayavattanakul M., Chongnativisit W., Chaikul P., Lourith N. (2020). Phenolic-Rich Pomegranate Peel Extract: In Vitro, Cellular, and In Vivo Activities for Skin Hyperpigmentation Treatment. Planta Med..

[24] Rasul A., Akhtar N. (2011). Formulation and in Vivo Evaluation for Anti-Aging Effects of an Emulsion Containing Basil Extract Using Non- Invasive Biophysical Techniques. Daru.

[25] Nichols J., Katiyar S. (2009). Skin Photoprotection by Natural Polyphenols: Anti-Inflammatory, Antioxidant and DNA Repair Mechanisms. Arch. Dermatol. Res..

[26] Zillich O.V., Schweiggert-Weisz U., Eisner P., Kerscher M. (2015). Polyphenols as active ingredients for cosmetic products. Int. J. Cosmet. Sci..

[27] Laguerre M., Lecomte J., Villeneuve P. (2007). Evaluation of the Ability of Antioxidants to Counteract Lipid Oxidation: Existing Methods, New Trends and Challenges. Prog. Lipid Res..

[28] Kusumawati I., Indrayanto G., Atta-ur-Rahman (2013). Chapter 15-Natural Antioxidants in Cosmetics. Studies in Natural Products Chemistry.

[29] Singleton V.L., Orthofer R., Lamuela-Raventós R.M. (1999). [14] Analysis of Total Phenols and Other Oxidation Substrates and Antioxidants by Means of Folin-Ciocalteu Reagent. Methods in Enzymology.

[30] Tuberoso C.I.G., Boban M., Bifulco E., Budimir D., Pirisi F.M. (2013). Antioxidant Capacity and Vasodilatory Properties of Mediterranean Food: The Case of Cannonau Wine, Myrtle Berries Liqueur and Strawberry-Tree Honey. Food Chem..

[31] Gyamfi M.A., Yonamine M., Aniya Y. (1999). Free-Radical Scavenging Action of Medicinal Herbs from Ghana: Thonningia Sanguinea on Experimentally Induced Liver Injuries. Gen. Pharmacol..

[32] Kallithraka S., Mohdaly A.A.-A., Makris D.P., Kefalas P. (2005). Determination of Major Anthocyanin Pigments in Hellenic Native Grape Varieties (*Vitis Vinifera* Sp.): Association with Antiradical Activity. J. Food Compos. Anal..

[33] Rodrigues Ueoka A., Pedriali Moraes C.A. (2018). Development and Stability Evaluation of Liquid Crystal-Based Formulations Containing Glycolic Plant Extracts and Nano-Actives. Cosmetics.

[34] Whangsomnuek N., Mungmai L., Mengamphan K., Amornlerdpison D. (2019). Efficiency of Skin Whitening Cream Containing Etlingera Elatior Flower and Leaf Extracts in Volunteers. Cosmetics.

[35] Stojiljković D., Tadić V., Stanković M., Roganović S., Arsić I. (2018). Standardized Extract of Wild Apple Fruit in Alkyl-Polyglucoside-Based Cosmetic Cream-Estimation of Stability, Safety, Antioxidant Activity and Efficiency. Int. J. Cosmet. Sci..

[36] Becker L.C., Bergfeld W.F., Belsito D.V., Hill R.A., Klaassen C.D., Liebler D.C., Marks J.G., Shank R.C., Slaga T.J., Snyder P.W. (2019). Safety Assessment of Glycerin as Used in Cosmetics. Int. J. Toxicol..

[37] Ferreira S.M., Santos L. (2022). A Potential Valorization Strategy of Wine Industry By-Products and Their Application in Cosmetics—Case Study: Grape Pomace and Grapeseed. Molecules.

[38] Ju Y., Yang L., Yue X., Li Y., He R., Deng S., Yang X., Fang Y. (2021). Anthocyanin Profiles and Color Properties of Red Wines Made from *Vitis Davidii* and *Vitis Vinifera* Grapes. Food Sci. Hum. Wellness.

[39] Sandhu A.K., Gu L. (2010). Antioxidant Capacity, Phenolic Content, and Profiling of Phenolic Compounds in the Seeds, Skin, and Pulp of Vitis Rotundifolia (Muscadine Grapes) As Determined by HPLC-DAD-ESI-MS(n). J. Agric. Food Chem..

[40] Vidal-Casanella O., Moreno-Merchan J., Granados M., Nuñez O., Saurina J., Sentellas S. (2022). Total Polyphenol Content in Food Samples and Nutraceuticals: Antioxidant Indices versus High Performance Liquid Chromatography. Antioxidants.

[41] Kosuru R.Y., Roy A., Das S.K., Bera S. (2018). Gallic Acid and Gallates in Human Health and Disease: Do Mitochondria Hold the Key to Success?. Mol. Nutr. Food Res..

[42] Li C., Zhang L., Liu C., He X., Chen M., Chen J. (2022). Lipophilic Grape Seed Proanthocyanidin Exerts Anti-Cervical Cancer Effects in HeLa Cells and a HeLa-Derived Xenograft Zebrafish Model. Antioxidants.

[43] Mandic A.I., Đilas S.M., Ćetković G.S., Čanadanović-Brunet J.M., Tumbas V.T. (2008). Polyphenolic Composition and Antioxidant Activities of Grape Seed Extract. Int. J. Food Prop..

[44] Isemura M. (2019). Catechin in Human Health and Disease. Molecules.

[45] Butkeviciute A., Ramanauskiene K., Janulis V. (2022). Formulation of Gels and Emulgels with Malus Domestica Borkh: Apple Extracts and Their Biopharmaceutical Evaluation In Vitro. Antioxidants.

[46] Zhao Z., Moghadasian M.H. (2010). Bioavailability of Hydroxycinnamates: A Brief Review of in Vivo and in Vitro Studies. Phytochem. Rev..

[47] Stanciu G., Lupsor S., Popescu A., Oancea I. (2022). Polyphenols Isolation and Determination in Grape Seeds by HPLC/DAD.

[48] Xia E.-Q., Deng G.-F., Guo Y.-J., Li H.-B. (2010). Biological Activities of Polyphenols from Grapes. Int. J. Mol. Sci..

[49] Soobrattee M.A., Neergheen V.S., Luximon-Ramma A., Aruoma O.I., Bahorun T. (2005). Phenolics as Potential Antioxidant Therapeutic Agents: Mechanism and Actions. Mutat. Res..

[50] Zhou K., Raffoul J.J. (2012). Potential Anticancer Properties of Grape Antioxidants. J. Oncol..

[51] Pinto D., Lameirão F., Delerue-Matos C., Rodrigues F., Costa P. (2021). Characterization and Stability of a Formulation Containing Antioxidants-Enriched Castanea Sativa Shells Extract. Cosmetics.

[52] Ali Ishaq M. (2020). Formulation and In Vitro Evaluation of Cream Containing *Vitis Vinifera* Fruit Extract. Master’s Thesis.

[53] Rafique M., Shah N. (2019). Anti-Ageing Potential of a Cream (W/O Emulsion) Containing Grape Seed Extract (GSE): Formulation and in Vivo Evaluation of Effectiveness Using Non-Invasive Biophysical Technique. J. Clin. Exp. Dermatol. Res..

[54] Rafique M., Nisar S., Shah H., Hussain I., Javed I., Nisar N., Riaz R. (2021). Development of Grape Seed Extract Based Formulations by Using Non- Invasive Biophysical Technique and Its Impact on Skin Aging. Pak. J. Pharm. Sci..

[55] Ross C.F., Hoye C., Fernandez-Plotka V.C. (2011). Influence of Heating on the Polyphenolic Content and Antioxidant Activity of Grape Seed Flour. J. Food Sci.

[56] Vural N., Algan Cavuldak Ö., Anlı R.E. (2018). Multi Response Optimisation of Polyphenol Extraction Conditions from Grape Seeds by Using Ultrasound Assisted Extraction (UAE). Sep. Sci. Technol..

