# Supplementary figures and images for: Stability and Antioxidant Activity of Hydro-Glyceric Extracts Obtained from Different Grape Seed Varieties Incorporated in Cosmetic Creams
Source: Antioxidants (Basel). 2022 Jul 10;11(7):1348. doi: 10.3390/antiox11071348 (PMC9311904; doi:10.3390/antiox11071348)

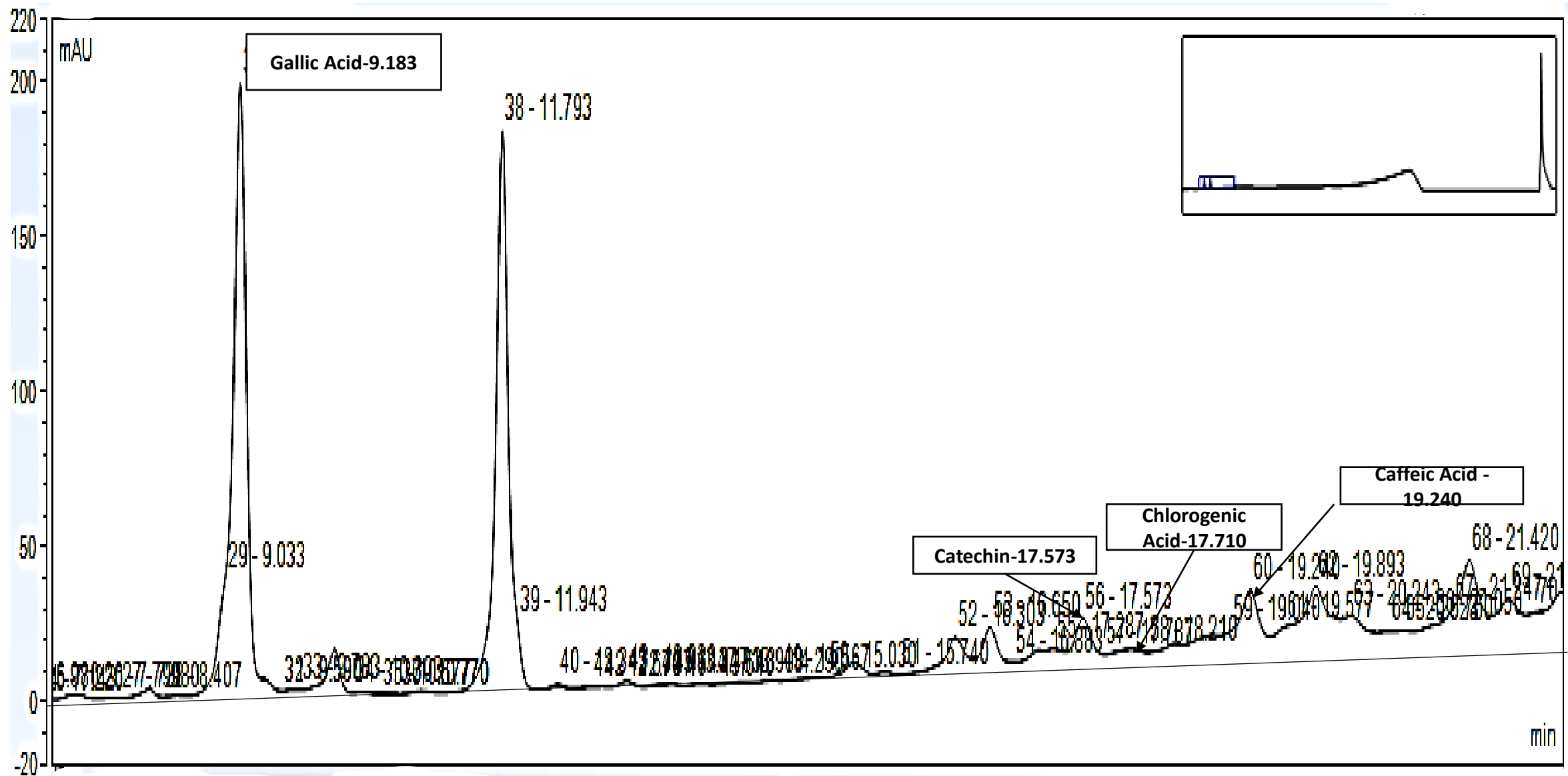

Figure S1 : Representative chromatogram of the performed HPLC analysis

Supplement: Supplementary file 1 [file antioxidants-11-01348-s001.zip › antioxidants-1782759-supplementary.pdf]
